# Annotations

**Published:** 1910-08-06

**Authors:** 


### ANNOTATIONS.

### Virchow's Monument.

After much delay, caused by differences between the sculptor and the scientific committee formed to carry out the work, the monument which Berlin has dedicated to the memory of Rudolph Virchow was unveiled on June 29. The ceremony, which took place on the Karlsplatz, in the heart of the university world, was of the simplest. A discourse on the great merits of Virchow as a scientist, as a politician, and as a philanthropist was read in the presence of his widow, his children, his colleagues, and the municipal authorities. As the veil fell it revealed an imposing group in limestone representing the triumph of a Titan over a monster, and on the base of the monument a life-like marble medallion of the deceased scientist. The group and portrait are by the sculptor Fritz Klimsch.

### A Dental Aid Experiment.

The interest which was excited in both public and professional circles by the announcement, made a few months ago, that the sum of £200,000 had been offered for the purpose of founding dental clinics on a self-supporting basis (see The Hospital, April 16, 1910, p. 69), has again been aroused to an even greater degree by the first practical step to test the working of the scheme. On July 6 a small dental clinic was opened in London in charge of two registered and qualified dental surgeons. This was announced in the daily Press, and the object was also widely circulated—namely, the benefit of the deserving poor who can, and will, pay small fees for dental assistance, but cannot afford the usual charges of the dental profession. From the paragraphs which have subsequently appeared in the papers it would seem that this experimental clinic has been highly successful in reaching just those classes for whose benefit the larger scheme has been devised. It also turns out, according to the *Daily Chronicle*, that this preliminary experiment is a private venture financed by Mr. Rayner, who is the Secretary of the British Dental Association, but this gentleman expressly disclaims any official connection between the Association and the new clinic. Indeed, it would seem that, though no profit is looked for, the experiment is intended to pay its way, and that if this cannot be done the clinic will have to be shut up. Whether such dental aid can be supplied without abuse by persons who are able, but unwilling, to pay adequate professional fees remains to be seen. There can be little question that the initial experiment has answered its purpose already, and has satisfied those who have instituted it of the reality of the demand. It is further contemplated that a benevolent fund shall be established, for which it is hoped to obtain a generous public support, to assist those patients who are unable to pay even the low fees which will be charged at the new clinics. This fund will be administered by a practical working committee, composed of members nominated by the Charity Organisation Society and similar bodies. It is proposed to give free treatment and artificial appliances only in special cases; preference will be given to those able to pay part of the cost of treatment. It is claimed, with considerable show of reason, that the working effect of this truly benevolent scheme will tend to lighten the labours of those who are working in the fields of tuberculosis and hygiene.

### The Signs of Death.

An article by Hereward Carrington in the *Annals of Psychical Science*, under the heading "Death: its Phenomena," reminds us that no really reliable proof of death, apart from that afforded by the onset of putrefactive processes in the body, has as yet been discovered. The author shows that a number of tests are known which, if applied in conjunction with each other and yielding positive results, may be taken as reasonable evidence of the onset of the condition which we call death, but that there is no simple test which, when put into action by human agency, will yield definite and unmistakable proof that life has left the body. This limitation to the powers of diagnosing a condition which sooner or later will overtake us all is not the result of apathy or idleness on the part of medical investigators. Much work has been done of late on the subject, both in this country and on the Continent, and every new discovery in science has been turned to the same purpose, of finding a test that will satisfy in all particulars the necessary requirements. Even the *x*-rays have been pressed into service to that end, but unfortunately without success. Whether the problem is insoluble time alone will show. Maybe, as the author remarks, death will never be understood until such time as we become acquainted with the true meaning of life and its nature. Meanwhile we have to content ourselves with the knowledge we already possess of the signs and symptoms of death, and no death-certificate should be filled up until the prospective writer of it has satisfied himself that death has occurred. The practice which is sometimes indulged in, especially in country districts where communication between doctor and patient is difficult and fees out of all proportion to the time and distance involved in journeying to the bedside of the supposed corpse, of filling in a certificate on the mere testimony of a relative or friend of the deceased, though not illegal as the law now stands, cannot be defended. The physician, and the physician alone, is the proper person to decide as to the onset of death, since he alone has had the training necessary to diagnose the condition with anything approaching certainty.

